# Medical News and Miscelleny

**Published:** 1888-04

**Authors:** 


### MEDICAL NEWS AND MISCELLANY.

---

WANTS THE NEXT MEDICAL CONVENTION.—The Waco Medical Society, by unanimous vote, decided to invite the Texas State Medical Association to hold its next meeting (1889) in Waco.

---

DR. J. H. MCLANE was elected City Physician of Fort Worth.

---

DR. JACOB PRICE, just from the halls of the famous Rush Medical College, has located at Rockland, Tyler county, and Dr. J. H. McCaleb, ditto from the ditto Tulane, has settled at Garfield.

---

REMOVALS.—Dr. C. O. Matthews, from Daingerfield to Belden; Dr. G. S. Holman from Schulenburg to Halfmoon; Dr. R. P. Tye from Iredell to Quanah.

---

STILL ANOTHER ORGANIZATION.—The physicians of Ellis county met in Waxahachie and organized on the 16th of March. Dr. J. C. Loggins was elected President, Dr. M. L. Haggard, Vice President, and Dr. L. Merriwether, Secretary.

---

DR. A. B. DELOACH, of Texarkana, let his pistol fall just as h was retiring for the night, and it was discharged, inflicting two painful wounds—one in the head and one in the hand.

---

FOR THE TEXAS INSANE.—The Superintendent of the Texas State Lunatic Asylum, Dr. J. S. Dorset, requests donations of books and papers to the embryo library which has been started for the benefit of the unfortunates under his charge. Deliver all donations to the Pacific Express Co., who will transport them free of charge. It is very gratifying to learn that Mrs. Frank Leslie has made a very handsome contribution of magazines and other light literature.

---

SERIOUS ACCIDENT TO DR. CLOPTON.—We regret to learn that on the 30th ult. Dr. A. G. Clopton, of Jefferson, one of the leading physicians of Texas, and a pillar in the State Association, was thrown from his buggy, suffering fracture of both bones of the right leg. It is especially to be regretted, as it will necessarily prevent his being present at the Galveston meeting, where he will be much missed. The JOURNAL extends sympathy to the Doctor, and ventures to express the hope that the injury may not be as serious as reported.

NEW DOCTORS—TFXAS AHEAD.—The Medical Department of the University of Louisville graduated one hundred new doctors on March 1st. Twenty-one of them were from Texas. The Texas boys captured the best prizes. William R. Spaulding, of Austin, was awarded the Yandell prize for the best class standing. Also the Anderson prize for Obstetrics, and the Bailey prize for Materia Medica, Therapeutics and Hygiene. Richard D. King and Joseph W. Largent, both of Texas, were awarded prizes, and August Schenk, of Texas, delivered the class valedictory. The Lone Star is proud of her sons, and the Capital City is doubly proud.

MR. CRUTCHFIELD, the affable agent and representative of Reed & Carnrick, of New York, has recently visited the Capital City. He ls the right man in the right place, and talks "Carnrick Soluble Food" and artificial digestion, with the fluency of a professor of dietetics. He is evidently making C. S. F. well known and popular in Texas.

DOCTORS AS MAYORS.—Dr. T. J. Largen was elected Mayor of Lampasas at last election. Dr. H. S. Broils was re-elected Mayor of Fort Worth—over strong competition; and Dr. J. Webb Douglass of Palastine was not exactly elected Mayor, but one of the Aldermen—next thing to it.

DOCTOR'S HOUSE BURNED.—We much regret to learn that the handsome residence of Dr T. J. Largen, at Lampasas, was entirely destroyed by fire on the 4th inst.,—caused, it is supposed, by a defective flue. Insurance only $2,000; loss $3,500.

DOCTOR'S SON MARRIED.—Mr. Montrose Burt, eldest son of the late popular Secretary of the Texas State Medical Association, Dr. W. J. Burt, was married at Austin on the 4th inst., to Miss Leontine Brunet.

MRS. VIVIAN, representing the manufacturing house of J. P. Bush & Co., of New York, visited Austin recently, introducing "Bovinine" to the medical profession. She is a cultivated lady of pleasing address, and presents the subject in such a fascinating way that it becomes a real pleasure to buy and try "Bovinine," the new fluid food for invalids. Mrs. Vivian brings letters from the leading physicians of other States, and we commend her to the courtesy of the profession in Texas.

A TABULATED report of Fourteen Cases of Calculus (cystic and urethral), by Prof. W. B. Rogers, of Memphis, will appear in the next issue of the JOURNAL.

DR. GABE FELDER has removed to Colmesneil, and Dr. Frank M. D. Hill, from Webberville to Dunbar, Travis county.

DR. J. S. LETCHER, of Lampasas, is at the Polyclinic, and like a number of others of our subscribers who don't want to be deprived even a short while, of the visits of the "Star" JOURNAL, requests it sent to him in New York, care of Prof. Wyeth, the courteous Secretary. (Wyeth is a Southern man—Arkansian, we believe—and was once a steamboat's physician, so we have heard. Alas, how are the mighty *risen!*

---

IT TELLS THE TALE.—FAIRCHILD, BROTHER & FOSTER, of New York, one of our whole-page advertisers, who have been with us from the beginning, paying an increased rate each year, writes us, under date, February 27, 1888:

"Our Mr. Holden, traveling in Texas, advises us that he believes that the bulk of the trade this house had in Texas, previous to his visit, was largely due to your efforts in our behalf, and to our advertisement in DANIEL'S TEXAS MEDICAL JOURNAL."

THE J. P. BUSH, MANUFACTURING CO., 2, Barclay street, New York, of whom we say as of the above—writes us as follows: Feb. 22, 1888.

"Your JOURNAL over and over again beats all the publications of the sort, south of Mason and Dixon's line, in enterprise and vigor, and is worthy of all the encomiums that are showered upon it; and you may well be proud of its great success."

[This firm has advertised in the JOURNAL two years, paying 50 per cent. advance the present year, in rate. It shows that advertising pays, or they wouldn't do it.]

THE SUBSCRIBERS, in remitting, say, about as follows—most of them something nice:

"I am much pleased with the JOURNAL, and must have it. Much success to you, and it; and all Texas physicians should subscribe for, and support so creditable a home journal." F. M. S.

"I find I cannot get along in my business without DANIEL'S TEXAS MEDICAL JOURNAL, so I send along the $2.00 for another year. May it ever continue to be what it is, and has been in the past,—*a true exponent of an honorable scientific profession.* Success to you!" Yours, etc.,

C. C. F.

---

SELF-CASTRATION.—Dr. R. H. Boland, in *Southern Medical Journal,* reports a case of self-castration, for seminal emissions. The patient recovered, and was *cured.*

